# Value of PET ECG gating in a cross-validation study of cardiac function assessment by PET/MR imaging

**DOI:** 10.1007/s12350-022-03105-2

**Published:** 2022-09-30

**Authors:** Alberto Villagran Asiares, Teresa Vitadello, Borjana Bogdanovic, Esteban Lucas Solari, Lachlan McIntosh, Sylvia Schachoff, Tareq Ibrahim, Stephan G. Nekolla

**Affiliations:** 1 Nuklearmedizinische Klinik und Poliklinik, Klinikum Rechts der Isar, School of Medicine, Technical University of Munich, Munich, Germany; 2 Klinik und Poliklinik für Innere Medizin I, Klinikum Rechts der Isar, School of Medicine, Technical University of Munich, Munich, Germany; 3 DZHK (German Centre for Cardiovascular Research), Partner site Munich Heart Alliance, Munich, Germany; 4 Department of Physical Sciences, Peter MacCallum Cancer Centre, Melbourne, Australia

**Keywords:** PET, MRI, hybrid imaging, viability, image analysis, diagnostic and prognostic application

## Abstract

**Background:**

This work investigated the impact of different cardiac gating methods on the assessment of cardiac function by FDG-PET in a cross-validation PET/MR study.

**Methods and results:**

MR- and PET-based left ventricular end-diastolic, end-systolic volumes, and ejection fraction (EDV, ESV, and EF) were delineated in 30 patients with a PET/MR examination. Cardiac PET imaging was performed using three ECG gating methods: fixed number of gates per beat (STD), STD with a beat acceptance window (STD-BR), and fixed gate duration (FW). High MR-PET correlations were found in all the values. ESVs correlated better than EDVs and EFs: Pearson’s *r* coefficient [0.92, 0.92, 0.92] in ESV vs [0.75, 0.81, 0.80] in EDV and [0.79, 0.91, 0.87] in EF, for each method [STD, STD-BR, FW]. Biases with respect to MRI for all the evaluated PET methods were less than 13% in EDV, 5% in ESV, and 14% in EF, but with wide limits of agreements, in the range (59-68)% in EDV, (65-70)% in ESV, and (49-71)% in EF. STD showed the strongest disagreement, while there were no marked differences between STD-BR and FW.

**Conclusion:**

Based on these findings, PET- and MR-based cardiac function parameters were highly correlated but in substantial disagreement with variabilities introduced by the selected PET ECG gating method. The most significant differences were associated with the ECG gating method susceptible to highly irregular beats, while similar performance was observed in the methods using uniform adjustment of gates width per beat with the beat acceptance window, and fixed gate width along all the beats. Thus, strict quality controls of R peak detection are needed to minimize its impact on the function assessment.

**Supplementary Information:**

The online version contains supplementary material available at 10.1007/s12350-022-03105-2.

## Introduction

The left ventricular (LV) cardiac function is routinely quantitatively assessed using several modalities of non-invasive cardiac imaging (MRI,^1^ echocardiography,^2^ SPECT^3^ and PET^4^). While MRI is considered by many (especially radiologists) the gold standard due to high temporal and intra-planar spatial resolution,^1^ the available resources in each hospital or health system and the patient condition (e.g., scanner, reimbursement system, claustrophobia, body size, pacemakers) determine the choice of the diagnostic modality in clinical routine. However, in-depth cross-validation analysis between the different modalities are required to improve the consistency of contractile ventricular function.

Quantitative assessment of LV cardiac function is performed by computing end-diastolic and end-systolic LV volumes (EDV and ESV), and the ejection fraction (EF). Cardiac gating techniques in cardiac imaging allow the temporal synchronization between the LV signal and the electrocardiogram (ECG) derived from the patient during the acquisition. This synchronization is used to assign the counts in a corresponding phase (gate) of the sampled cardiac cycle, obtaining later on an image per gate used to compute the LV volumes. However, technical and clinical issues such as long acquisitions, misdetection of R-waves,^5^ and arrhythmias lead to high cardiac cycle variability that might impair the gates formation, and ultimately the LV volumes accuracy.

Current PET scanners support cardiac gating imaging with list-mode data acquisition allowing retrospective definition of the cardiac phases,^6,7^ and thus, different gating approaches can be investigated to determine their impact on the cardiac function assessment. Moreover, hybrid PET/MR scanners with near simultaneous acquisitions can be employed as an enhanced cross-validation system. It is possible to measure the same parameters with PET and MR acquired in only one examination under almost identical physiological conditions and to compare them. This near simultaneous information potentially improves the multimodality comparison, reducing the impact of cofounders related to temporal/spatial misalignments (e.g., patient repositioning, time-dependent physiological conditions, and repeated breath holds during acquisition) in the cross-validation analysis.

Therefore, the objective of this work is to evaluate the impact of PET cardiac gating procedure on the assessment of LV cardiac function in an enhanced cross-validation multimodal study. We present the correlation and agreement analyses performed on the LV volumes and ejection fraction computed from MRI and from three cardiac gating PET alternatives, acquired in a near simultaneous PET/MR system. Additionally, we discuss the potential causes for the discrepancies between the modalities and compare results with previous cross-validation studies.

## Methods

### Demography

This study was performed in a cohort of 30 patients (demographic information in Table 1) with known chronic total occlusion previous to revascularization procedure to assess myocardial viability using an integrated PET/MRI. All subjects gave their written informed consent in accordance with the guidelines of the local ethics board that approved the study 169/16 S.

**Table 1:** Demographic summary of the patients’ cohort

N	30
Male sex	29 (97%)
Age (years)	66 ± 9
Body mass (kg/m^2^)	28 ± 4
Diabetes	7 (23%)
Hypertension	25 (83%)
Smoking	16 (53%)
Medication	Angiotensin-converting enzyme 28 (93%)
	Beta-blockers 26 (87%)
	Diuretics 12 (40%)
	Statins 29 (97%)
Dyslipidemia	23 (77%)
Family history	7 (23%)
Multivessel CAD	28 (93%)
Previous myocardial infarction	9 (30%)
Coronary artery bypass	5 (17%)
CTO Localisation	LAD 9 (30%)
	LCX 7 (23%)
	RCA 15 (50%)

*N*, number of subjects; *LAD*, left anterior descending artery; *LCX*, left circumflex artery, *RCA*, right coronary artery

### Imaging protocol

Imaging was performed using a simultaneous PET/MR system (Biograph mMR, Siemens Healthcare, Erlangen, Germany). ECG signal was recorded with MR-compatible 3-lead electrodes and used for both cardiac MRI acquisition and ECG-gated PET reconstructions.

### MR imaging

Conventional multi-slice 2-dimensional short axis CINE sequences were used to obtain the reference values of the volumes and the ejection fraction of the left ventricle at the beginning of the PET/MR exam. The images have a reconstructed matrix size: 256 × 208, number of slices: 10-13, voxel size: 1.33 × 1.33 × 8 mm^3^, spacing between slices: 8 mm, and temporal resolution: 25 cardiac phases.

### PET imaging

To optimize glucose uptake in the heart and to standardize the metabolic environment in all patients, a hyperinsulinaemic-euglycaemic clamp procedure was performed. After stabilization of the plasma glucose level for approximately 60 minutes, 330 ± 32 MBq of fluorine-18 fluorodeoxyglucose (FDG) (4 MBq prescribed per kg body weight) was administered intravenously. The electrocardiographic-gated (ECG gated) list-mode PET was started approximately 60 minutes after the intravenous injection of FDG. PET data were acquired during the whole PET/MR exam (average duration of 42 minutes with a range [40-50] minutes). PET images were reconstructed using e7tools framework (Siemens Healthcare, Knoxville, TN) with an ordinary Poisson ordered-subset-expectation maximization iterative reconstruction algorithm (OP-OSEM) with 3 iterations—21 subset, matrix size 344 × 344, zoom: 1, reconstructed voxel size: 2.08 × 2.08 × 2.03 mm^3^, and 8 cardiac phases. Attenuation correction maps were generated from a Dixon-based MRI sequence under breath-hold at end-expiration. Additionally, the maximum-likelihood reconstruction of attenuation and activity (MLAA) algorithm was used to correct for arm truncations in the Dixon attenuation maps occurring due to the limited MRI field-of-view.^8^

### PET cardiac gating methods

ECG-based cardiac gating with 8 phases was performed on PET list-mode data. Cardiac cycles (hereafter R-R intervals) were considered as the time between two consecutive R peaks detected by the PET/MR system. Each R-R interval was divided into gates and the PET counts acquired in each one were grouped to reconstruct an image per gate.

**Figure 1:**
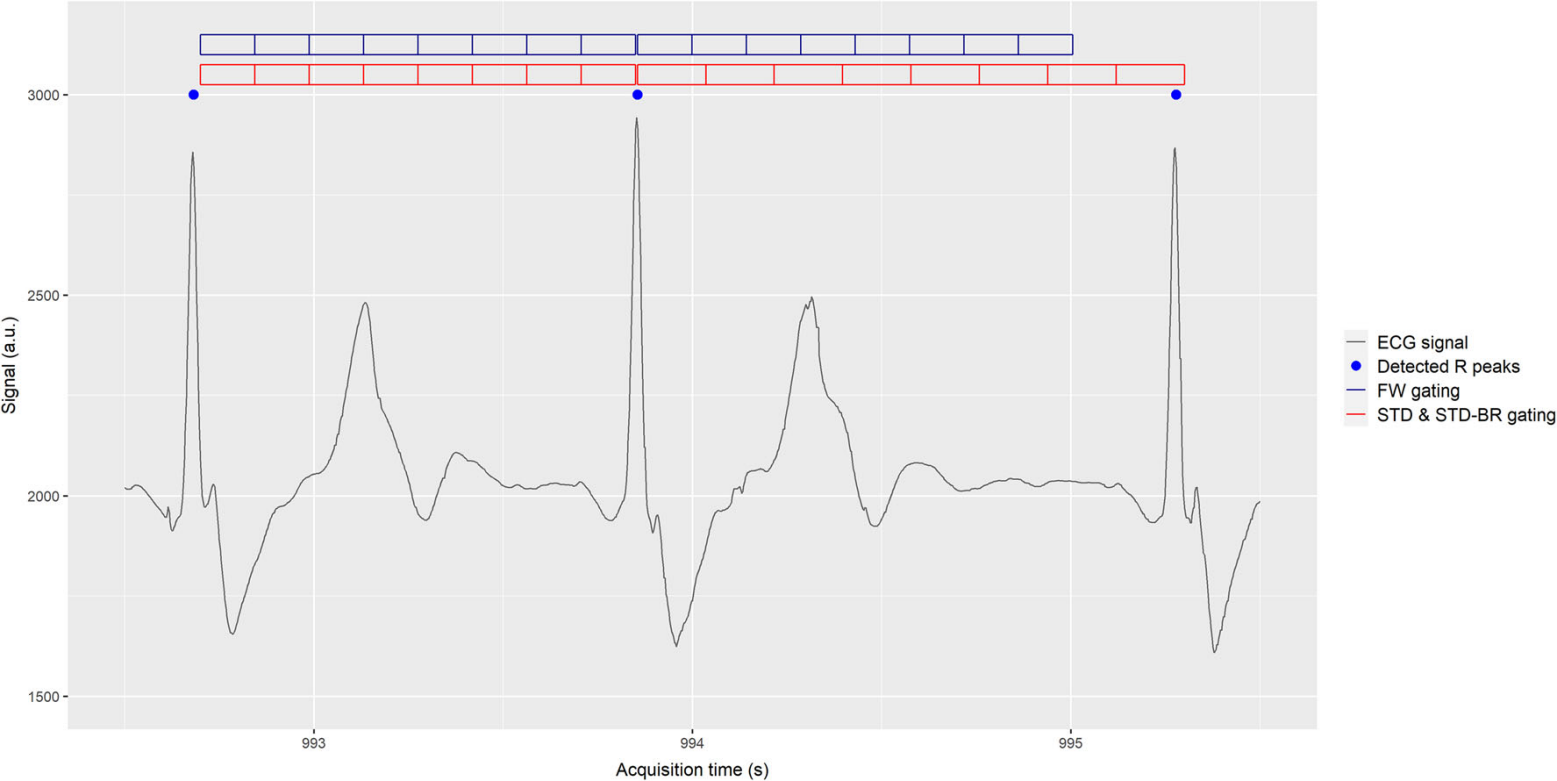
ECG gating methods for cardiac PET imaging. ECG signal sample measured during the exam (black line) with the detected R peaks by the PET/MR system (blue dots). Cardiac phases definition (gates) based on two approaches: fixed gate duration (FW gating [upper blue grid]), and fixed number of gates (STD and STD-BR gating [lower red grid]) per cardiac cycle

**Figure 2:**
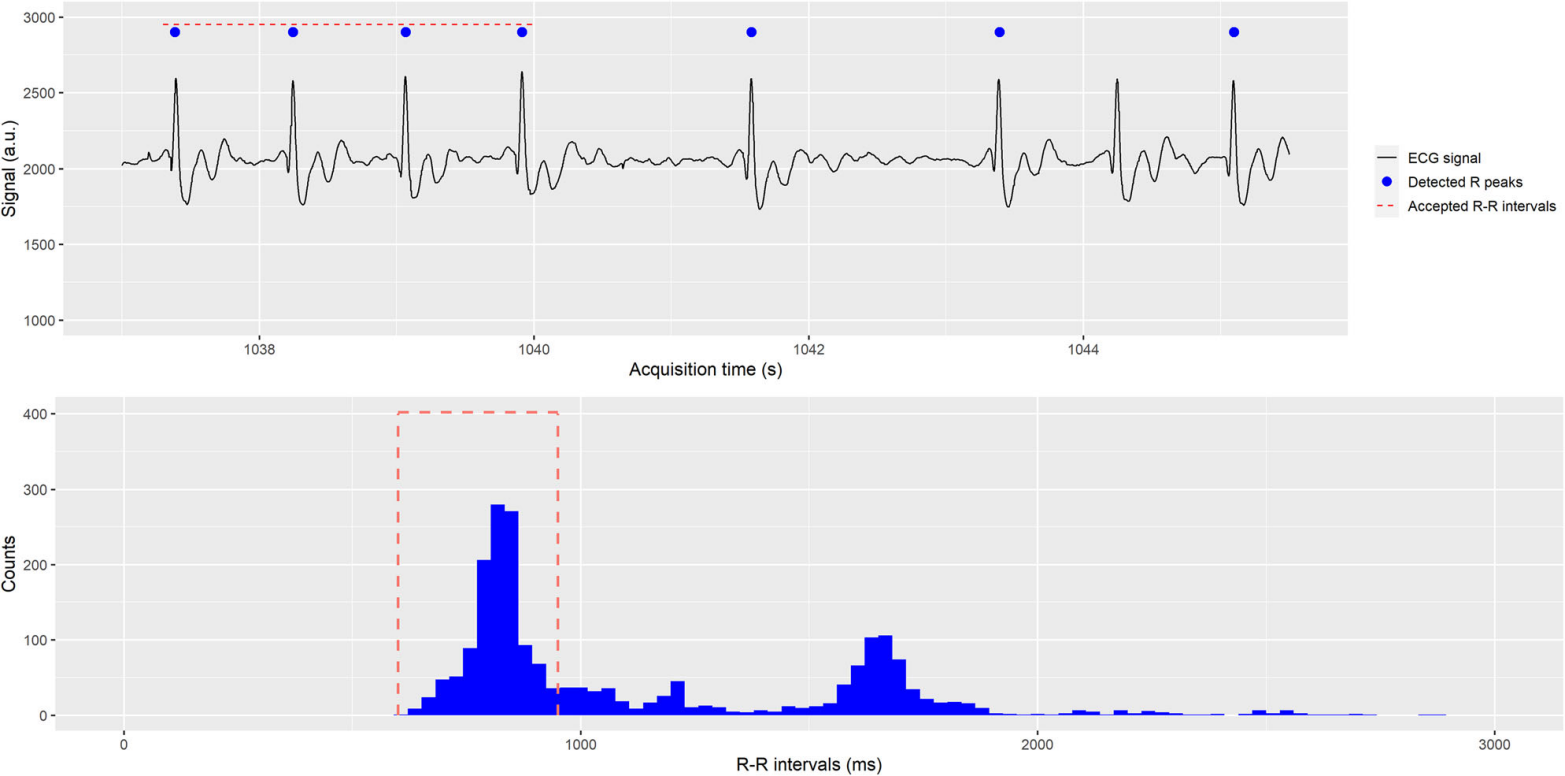
Upper row: ECG signal (black line) sample with the detected R peaks by the PET/MR system (blue dots) and R-R intervals considered by the acceptance window (red dash line). Abnormal R-R intervals (longer R-R intervals due to arrhythmias and artefacts in R peak detection) are not used in PET reconstruction in STD-BR ECG gating method. Lower row: R-R intervals histogram with the selected window acceptance (dash red line) used in STD-BR

Three cardiac gating methods were studied (Figure 1):

1. Standard gating (STD) keeps constant the number of gates per R-R interval dividing each cycle into exactly 8 gates.
2. Beat rejection gating (STD-BR) is similar to STD but it also rejects the abnormal R-R intervals generated by arrhythmias or artefacts in R peak detection (Figure 2). An acceptance window was manually defined on the R-R intervals distribution for each patient.
3. Fixed width gating (FW) preserves the duration of each gate along the whole acquisition. It defines a single gate width as 1/8 mean value of the distribution of R-R intervals (after abnormal beats rejection), and then extracts from each R-R interval up to 8 gates based on the single gate width.

The cardiac gating methods STD and STD-BR correspond to the default approaches available in the PET/MR, while FW addresses the physiological finding that the systolic phase remains constant among different cardiac cycles.^9^

### Volume measurements

**Figure 3:**
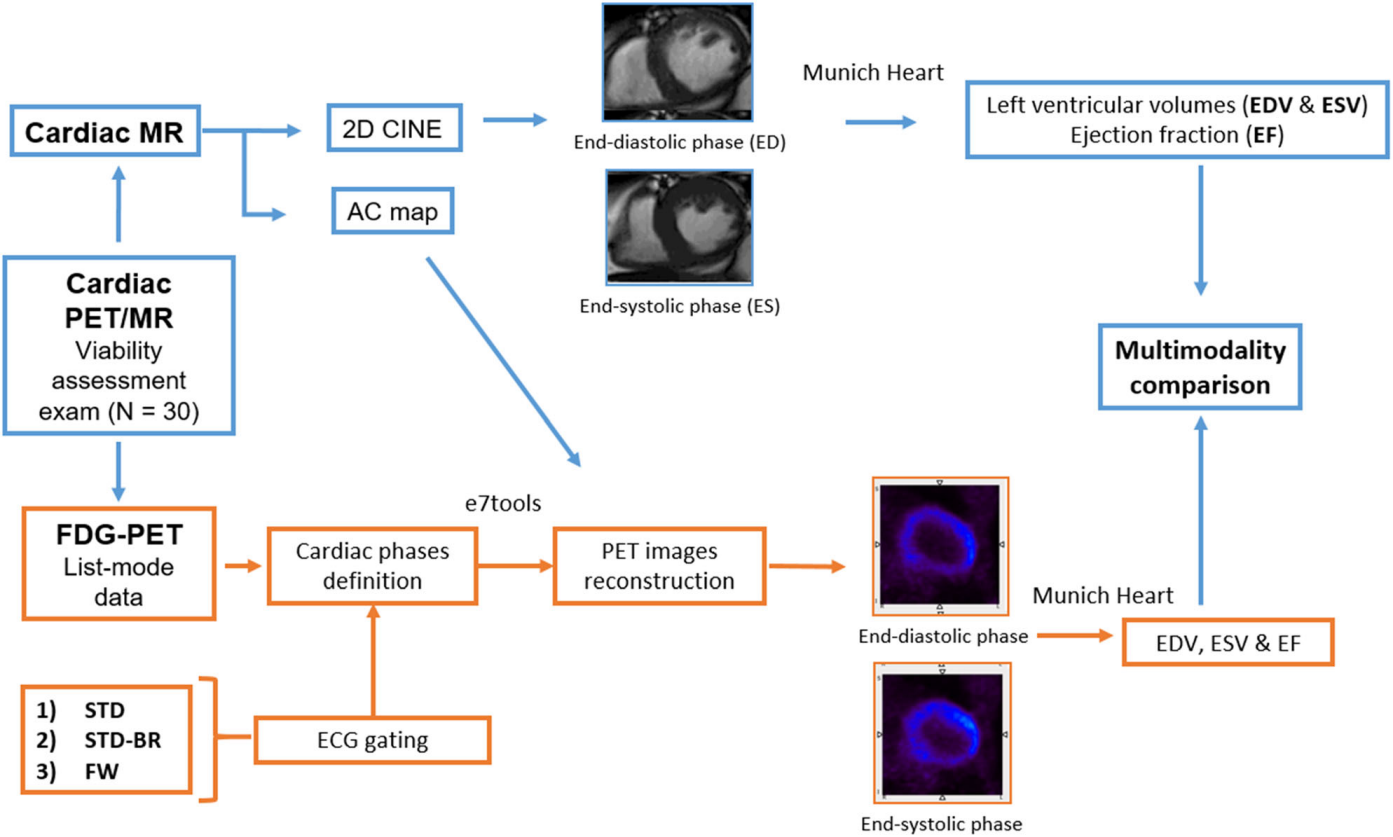
General scheme of data acquisition, cardiac PET reconstruction and PET/MR comparison of left ventricular volumes and ejection fraction. *Cardiac MR*, cardiac magnetic resonance; *PET*, positron emission tomography; *AC*, attenuation correction; *ECG*, electrocardiogram; *EDV*, end-diastolic volume; *ESV*, end-systolic volume; *EF*, ejection fraction; *LV*, left ventricle; *FDG*, Fluorine-18 fluorodeoxyglucose; *STD*, standard ECG gating method; *STD-BR*, standard - beat rejection ECG gating method; *FW*, fixed width ECG gating method

Volume measurements and analysis workflow are presented in Figure 3. End-diastolic and end-systolic volumes (EDV and ESV) as well as the corresponding left ventricular (LV) ejection fraction (EF) were assessed in both modalities using the software MunichHeart (MH).^10^ For 2-dimensional CINE images, the LV chamber was manually delineated by a cardiologist following standard guidelines.^1^ EDV and ESV were defined as the maximum and minimum values of ventricular volumes measured from phases that were visually defined as end systole and end diastole. Papillary muscle volumes were considered part of the LV chamber.

To analyze ECG-gated FDG-PET images, MH computed EDV and ESV using a geometric model based on regional uptake threshold of the myocardium using a manual definition of the long axis and the base plane of the LV. End-diastolic and end-systolic phases were defined automatically based on the PET counts in the cardiac cycle.^10^

### Statistics

The multi-modality comparison of EDV, ESV and EF from MRI and PET was performed via correlation analysis using Pearson’s *r* coefficient and linear regression [Deming’s method, considering the errors for the values of both modalities]), and via agreement analysis with a paired Wilcoxon test for mean comparison, and bias and limits of agreement of Bland-Altman was calculated with *stats*, *deming, and blandr* R packages (version 4.0.4, The R Foundation for Statistical Computing, Vienna, Austria). Differences were considered as statistically significant when corresponding statistic test presented a *P *value < .05.

## Results

### Abnormal R-R intervals statistics

In 8/30 subjects, the number of abnormal R-R intervals was greater than 10%, reaching up to 40% of the total R-R intervals detected by the scanner. Thus, as the STD-BR method rejected those, the effective exam time discarded in these subjects was on average 15 minutes (range [8-28] minutes) from the total PET acquisition durations of 43 minutes (range [40-50] minutes). There was no correlation between the body mass index of each subject and the amount of abnormal R-R intervals (Pearson’s *r* = − 0.08).

## Quantitative assessment of global cardiac function by MR and PET

### Correlation analysis

**Figure 4:**
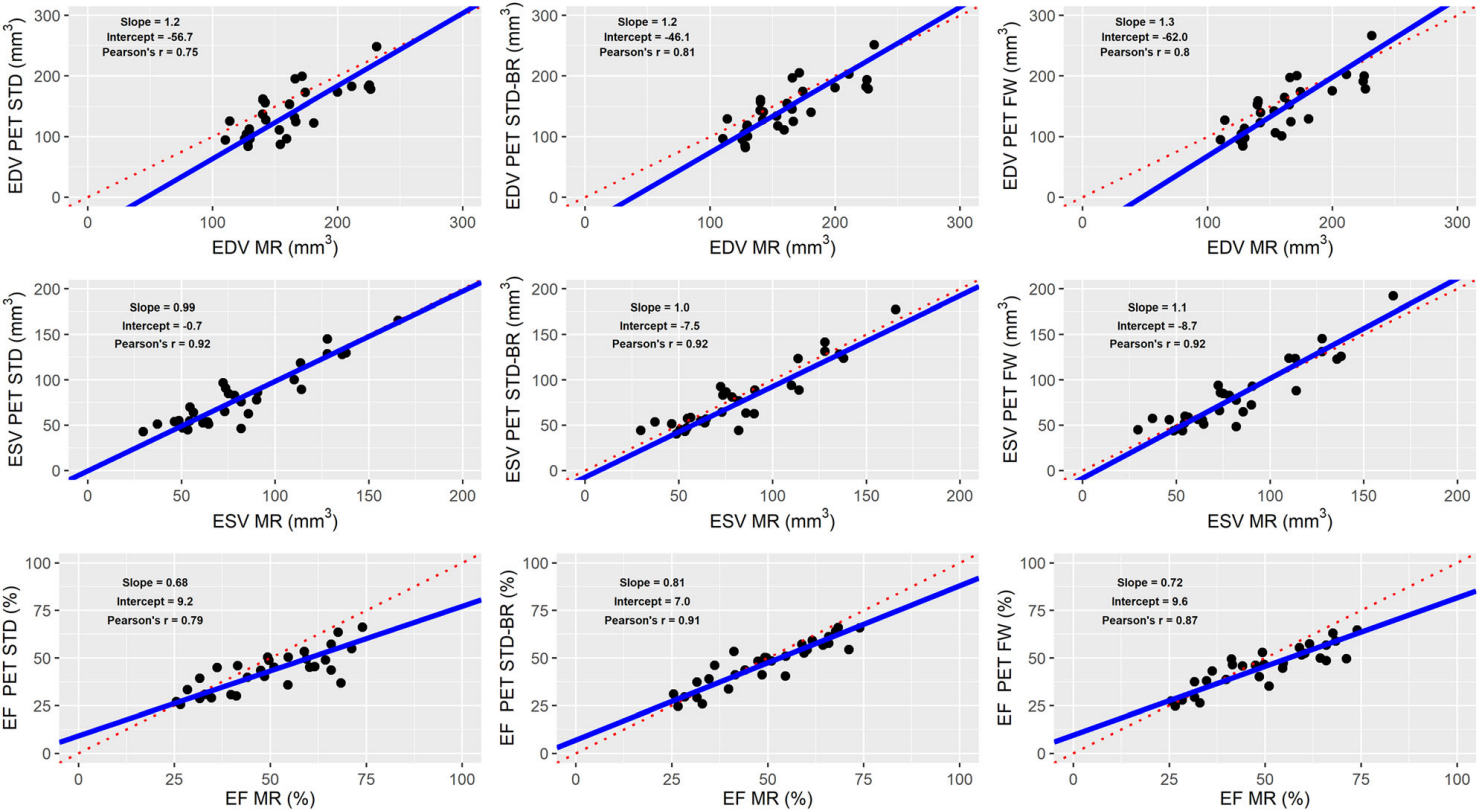
Correlation analysis of LV volumes and ejection fraction obtained with MR and PET. PET ESVs correlated better with MRI than PET EDVs. In overall, there were high correlations (Pearson’s *r* > 0.75), with STD-BR method with the best while STD yielded the poorest correlation. Identity line in red dashed line, and linear regression model in blue solid line

MRI- and PET-derived volumes as well as EF values presented overall high correlations (Figure 4). In the case of EDV, the slopes were 1.23 ± 0.18 (Pearson’s *r* = 0.75) for STD, 1.19 ± 0.15 (*r* = 0.81) for STD-BR, and 1.29 ± 0.16 (*r* = 0.80) for FW. In ESV, the slopes were 0.99 ± 0.08 (*r* = 0.92) for STD, 1.05 ± 0.08 (*r* = 0.92) for STD-BR, and 1.10 ± 0.09 (*r* = 0.92) for FW. Furthermore, in the case of EF, the slopes were 0.68 ± 0.09 (*r* = 0.79) for STD, 0.81 ± 0.07 (*r* = 0.91) for STD-BR, and 0.72 ± 0.07 (*r* = 0.87) for FW. Hence, PET ESVs correlated better with MRI than PET EDVs, and in particular, the STD-BR method yielded the best while STD yielded the poorest correlation.

### Agreement analysis

The comparison of mean values against MRI (Table 2) showed that PET imaging underestimated all the parameters, with statistically significant differences in EDV for all gating methods, and for EF also STD and FW.

**Table 2:** Mean values of end-diastolic and end-systolic volumes (EDV and ESV) and corresponding ejection fractions (EF) for the modalities MR, and PET reconstructed with the three methods of cardiac ECG gating: STD: standard ECG gating method, STD-BR: standard - beat rejection ECG gating method, FW: fixed width ECG gating method

Modality	EDV (mL)	ESV (mL)	EF (%)
MR	160 ± 35	82 ± 33	50 ± 14
PET-STD	140 ± 41*	80 ± 33	43 ± 10*
PET-STD-BR	145 ± 40*	78 ± 35	47 ± 11
PET-FW	145 ± 43*	81 ± 37	45 ± 10*

*Stands for statistically significant differences against MR values (*P *value < .05)

**Figure 5:**
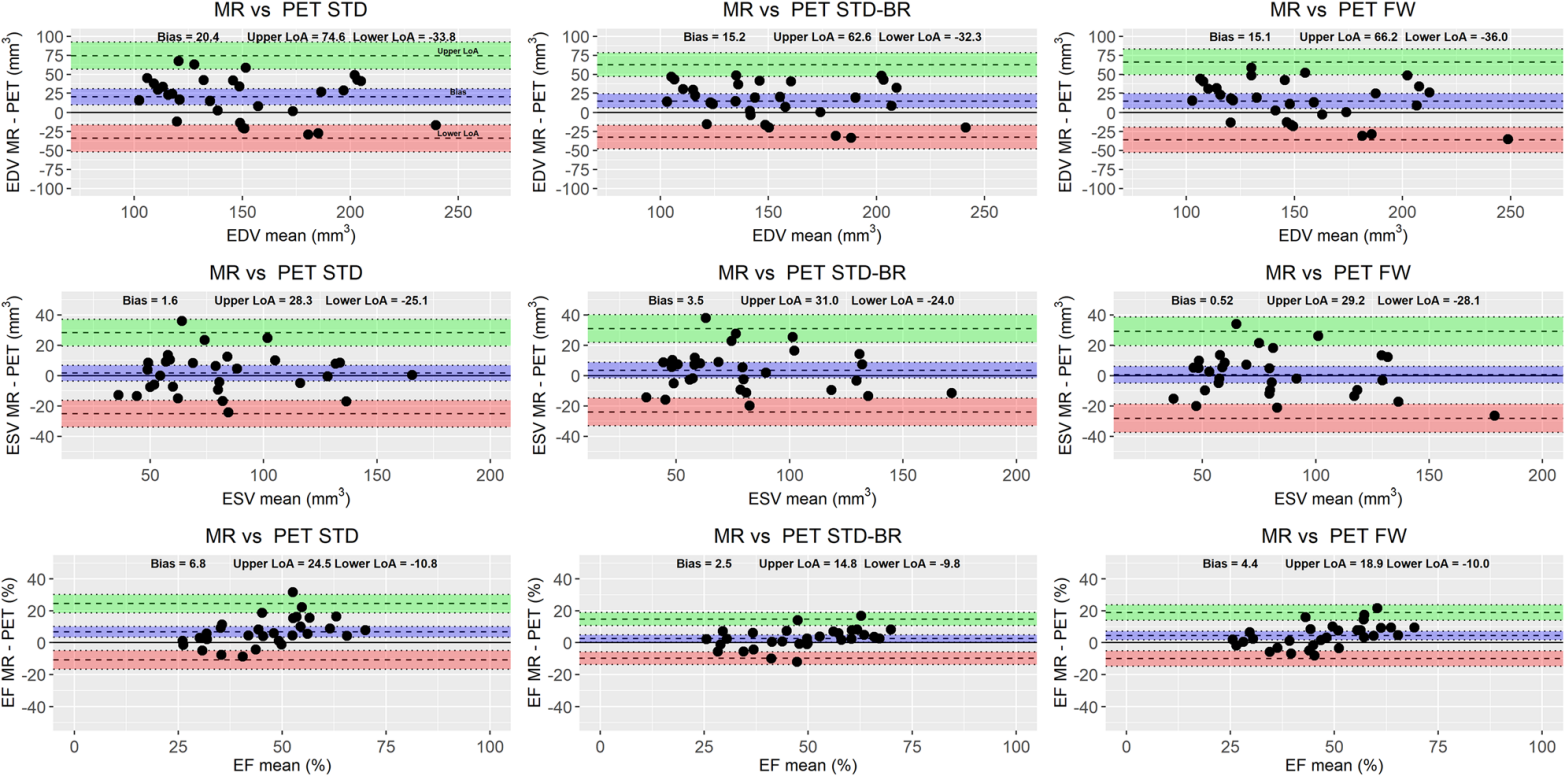
Bland–Altman plots of LV volumes and ejection fraction obtained with MR and PET modalities. There were slight to moderate underestimation by PET with wide limits of agreement. STD showed the strongest disagreement. No marked differences between STD-BR and FW methods

Comparison of Bland-Altman plots of volumes and ejection fraction obtained with MRI and PET are presented in Figure 5. The mean relative biases for the three methods in EDV were in the range (9-13)% with wide relative limits of agreement (60-68)%. For ESV, relative biases were much less pronounced (− 1-3)%, with similar limits of agreements (65-70)%. The range of EF relative biases was (5-14)% with limits of agreement (49-71)%. Here, there were no marked differences between the STD-BR and FW methods. However, STD showed the strongest disagreement.

## Discussion

This work studied the influence of PET cardiac gating on the LV cardiac function assessed from hybrid PET/MR exams of patients with known coronary disease. Correlation and agreement analyses were performed on LV contractile parameters obtained by cardiac MRI and PET images reconstructed with three gating methods. Using MR values as reference, PET-based values showed high correlation, slight to moderate trend toward underestimation, and wide limits of agreement. The most significant differences were found in the gating method that did not perform well in the setting of large R-R intervals variability (STD), while a similar performance was observed in the methods using uniform adjustment of gates width per beat with the beat acceptance window (BR-STD), and fixed gate width along all the beats (FW).

### Cardiac gating

Results from the STD gating approach showed the importance of the beat acceptance window. The inclusion of irregular R-R intervals in the gating process drives to merging PET counts from dissimilar cardiac phases, leading to an effective smoothing of the myocardium and subsequently affecting the estimation of left ventricular volumes. The similar performance of BR-STD and FW in this study indicated that the effects associated with the selection of the gate width could not be clearly discerned, potentially due to the higher impact of differences linked to the multimodal comparison PET-MRI, as discussed below.

Even though the STD-BR method avoids abnormal R-R intervals, the rejection of PET counts increases image noise, affecting the accuracy of the measurement. For instance, in one of the subjects, only 12 out of 40 minutes was used to reconstruct the PET image due to 40% of abnormal R-R intervals (equivalent to rejection of 66% of the total time)—an effect which would be even more detrimental in shorter exams. Extra efforts are needed to reduce this loss in counts: exhaustive quality controls to the ECG signal and R peak detected during acquisition, improvements of R peak detection algorithms (in particular, incrementing robustness to MR artefacts^11,12^), generalization of ECG gating taking thoughtfully into account arrhythmic heartbeats as well.

In particular, it is important to emphasize the need for a strict quality control of the ECG signal and its processing in the clinical environment, since these artefacts affect not only the cardiac PET gating method, but also the acquisition of the cardiac MRI sequences that use this signal as a trigger.^12^ Further investigations for alternatives to ECG-based cardiac gating are also encouraged.^13,14^

### Multimodal cross-validation

Comparable works^15–20^ studied FDG-PET and MRI-based values of EDV, ESV and EF obtained using PET/MR, PET/CT, PET and MRI systems. Table 3 summarizes the main results. In general, we can see high correlations, but varied biases: (− 1.1-28.4)% for EDV, (− 5.9-29.6)% for ESV, and (− 12.0-13.6)% for EF, and principally wide limits of agreement: (44.3-95.4)% for EDV, (25.5-153.9)% for ESV, and (26.1-142.5)% for EF. Another comparative study^21^ between nitrogen-13 ammonia PET and MRI-based values reported higher correlations and lower biases, but the limits of agreements are still wide (33.3% for EDV, 48.2% for ESV, and 29.7% for EF). These comparisons support the evidence that cardiac function measurements are not interchangeable between modalities.

**Table 3:** Comparison of correlation and agreement analyses in similar studies

Study	N	Scanner	Software PET	Software MR	# gates	EDV			
						Linear Slope	Pearson's r	Bias (%)	LoA amplitude (%)
Khorsand 2003	20	PET	In-house	Phillips	8	0.6	0.92	10.1	95.4
Schäfer 2004	42	PET	QGS	Phillips	8	1.0	0.94	0.3	44.3
			4D-MSPECT			1.0	0.94	− 1.1	46.6
Slart 2004	38	PET/CT	QGS	MASS	16	0.9	0.91	15.0	56.0
Li 2014	89	PET/CT	QGS	MASS	8	0.9	0.92	12.7	65.9
			4D-MSPECT			1.1	0.93	1.0	66.6
Lücke 2017	29	PET/MR	Corridor4DM	cmr42	16	1.0	0.95	16.5	62.3
Yao 2019	76	PET/CT	QGS	MASS	8	0.8	0.91	28.4	70.6
			ECTB			0.8	0.86	25.5	88.4
			4D-MSPECT			0.9	0.89	18.5	80.2
Our study STD	30	PET/MR	Munich Heart	Munich Heart	8	1.2	0.75	12.8	67.8
Our study STD-BR						1.2	0.81	9.5	59.3
Our study FW						1.3	0.80	9.4	63.9

Study	ESV				EF			
	Linear Slope	Pearson's r	Bias (%)	LoA amplitude (%)	Linear Slope	Pearson's r	Bias (%)	LoA amplitude (%)
Khorsand 2003	0.6	0.93	9.8	153.9	0.6	0.85	6.5	85.2
Schäfer 2004	1.0	0.95	− 5.9	25.5	0.7	0.94	11.4	51.4
	1.0	0.95	− 4.2	55.1	0.7	0.90	2.9	57.1
Slart 2004	0.9	0.94	13.7	58.2	1.0	0.96	10.3	26.1
Li 2014	0.9	0.92	11.7	83.4	0.9	0.76	0.3	115.3
	1.0	0.94	− 4.4	80.7	0.7	0.75	12.5	108.6
Lücke 2017	1.2	0.97	19.6	104.8	1.0	0.91	− 4.1	68.8
Yao 2019	0.9	0.93	28.1	77.8	1.0	0.79	− 3.6	113.8
	0.7	0.85	29.6	108.0	0.7	0.62	− 12.0	142.5
	0.9	0.91	18.1	92.6	0.8	0.76	0.7	105.8
Our study STD	1.0	0.92	2.0	65.1	0.7	0.79	13.6	70.6
Our study STD-BR	1.0	0.92	4.3	67.1	0.8	0.91	5.0	49.2
Our study FW	1.1	0.92	0.6	69.9	0.7	0.87	8.8	57.8

FDG-PET- and MR-based values of EDV, ESV, and EF of N number of subjects were obtained using PET/MR, PET/CT, PET, and MR systems, different MR and PET software, and 8 or 16 gates. MR and PET presented high correlations, variable biases, and wide limits of agreements (LoA) among the studies

Although such a setting should ideally be the perfect scenario for multimodal comparisons with comparable physiological conditions,^22^ several multiple factors contribute to the observed variability. In addition to potential intra-/inter-observer variability, the discrepancies between the two modalities might be also explained by temporal and spatial resolutions, differences in the geometrical model of the heart between MRI and PET, and volume variation with heart rate changes.

Since the effective number of PET gates is three times less than in MRI, the measured PET-derived LV volumes are potentially undersampled (smoothing effect), affecting the accuracy of the parameters. Studies compared the volumes obtained with different numbers of gates (8, 16, and 32) in SPECT and PET images^23–25^: when using only 8 gates, smaller EDV, larger ESV, and a corresponding lower EF was observed, with the highest impact found in ESV measurement (median changes among those studies of 3% vs − 10%, in EDV and ESV, respectively). Nevertheless, temporal resolution issues might not be sufficient to explain the discrepancies in our cohort since the highest differences were found in EDV values.

The delineation of short and long axes, of base and apex of the heart in both modalities, and the lack of compatibility between them may also increase variability in the definition of the geometric model used to measure LV volume. In MRI, the axes are defined prospectively before the acquisition of the cine images, while in PET, the task is retrospectively carried out by physicians as a part of the data post-processing, adding to the inter-observer variability from two fully different directions. Another plausible factor of the discrepancies is an inaccurate FDG uptake-based PET contouring due to the severity myocardial damage, however, most of the LV myocardial segments of this cohort were classified as viable by FDG-PET assessment.

The lack of a perfect time matching (different time and duration) between PET and MRI acquisition in the exam (total scan time in PET vs several minutes in MRI) potentially leads to parameters that may not fully coincide. Two studies^26,27^ analyzed the heart rate dependency of LV volumes of cardiac phantoms in SPECT/CT and CT images and a range 40-100 beats per minute. They found differences in EF of up to 2.5% in the CT images with low temporal resolution (175 ms per gate) between 60 and 80 beats per minute, but on average of only 1% with higher temporal resolutions (75 ms in CT and 38 ms in SPECT per gate). Regarding our study, even though PET temporal resolution was on average 120 ms (62 beats per minute), the spatial resolution was lower than with CT images, and thus intra-scan heart rate variation might be considered as part of the source for the discrepancies.

Even though the current clinical CINE MRI sequences provide images with the highest in-plane spatial and temporal resolutions, clinical 2-dimensional acquisition schemes limit the accuracy of inter-plane information. Due to the sequential, slice-by-slice acquisition, 2-dimensional images are more susceptible to motion, and the thickness of slices is acquired with worse spatial resolution than in the in-plane situation to cover the entire LV chamber in a feasible clinical acquisition time. Consequently, the use of cine MR values as the reference should be re-evaluated. In this sense, 3-dimensional acquisition for cine images seems to be the next step. However, clinically suitable schemes for its implementation are still in development and might hold other limitations in store.^28^

## Conclusion

This work investigated the impact of different cardiac gating methods on the assessment of the cardiac function assessed by FDG-PET in a cross-validation simultaneous PET/MR study.

PET and MRI parameters were highly correlated with a slight to moderate trend toward underestimation, and wide limits of agreement, presenting fluctuations depending on the PET ECG gating method. The most significant differences were associated with the ECG gating method susceptible to highly irregular beats, while similar performance was observed in the methods using uniform adjustment of gates width per beat with the beat acceptance window, and fixed gate width along all the beats.

It is highly recommended that a strict quality control of R peak detection is performed on a patient-by-patient basis to minimize its impact on the quantitative assessment. Moreover, this study once more confirms that the PET and MRI parameters are clinically not interchangeable and that the concept of multimodal cross-validations needs to be considered very carefully.

## New knowledge gained

LV contractive function assessments by FDG-PET and by MR imaging acquired near simultaneously showed strong correlations but poor agreements, with variabilities introduced by the ECG cardiac gating methods. Efforts for rigorous ECG gating quality control are encouraged in clinical practice.

## Supplementary Information

Below is the link to the electronic supplementary material.

Supplementary file1 (PPTX 555 kb)

Supplementary file2 (MP3 6946 kb)

## Abbreviations

PET: Positron emission tomography
MRI: Magnetic resonance imaging
SPECT: Single photon emission computed tomography
CT: Computed tomography
ECG: Electrocardiogram
EDV: End-diastolic volume
ESV: End-systolic volume
EF: Ejection fraction
LV: Left ventricle
FDG: Fluorine-18 fluorodeoxyglucose
STD: Standard ECG gating method
STD-BR: Standard - beat rejection ECG gating method
FW: Fixed width ECG gating method
