# Supplementary material for: Value of PET ECG gating in a cross-validation study of cardiac function assessment by PET/MR imaging
Source: J Nucl Cardiol. 2022 Sep 30;30(3):1050–60. doi: 10.1007/s12350-022-03105-2 (PMC10261229; doi:10.1007/s12350-022-03105-2)
Supplement: Supplementary file 1 — Supplementary file1 (PPTX 555 kb) [file 12350_2022_3105_MOESM1_ESM.pptx]

## Slide 1
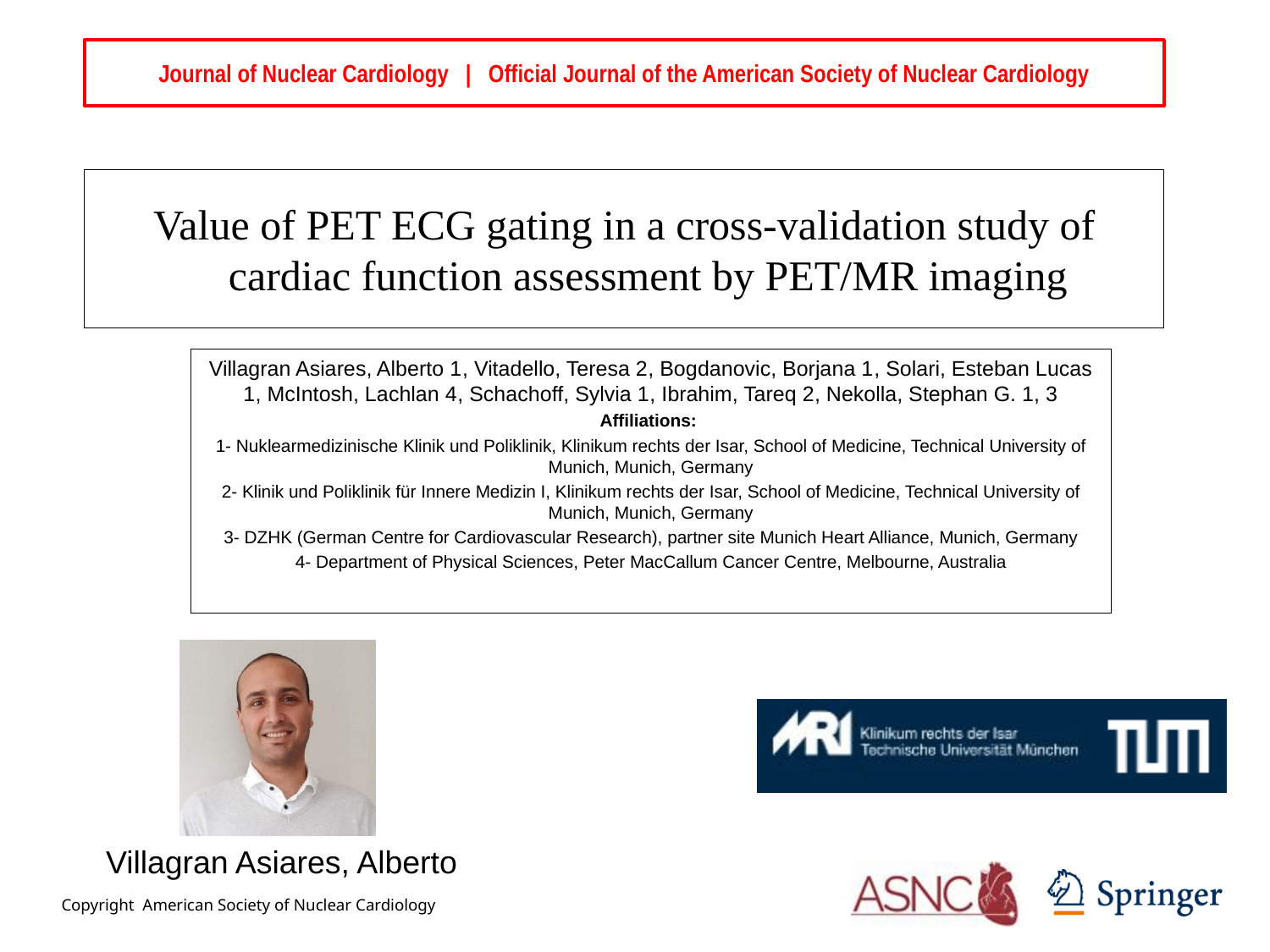

Journal of Nuclear Cardiology | Official Journal of the American Society of Nuclear Cardiology
# Value of PET ECG gating in a cross-validation study of cardiac function assessment by PET/MR imaging
Villagran Asiares, Alberto 1, Vitadello, Teresa 2, Bogdanovic, Borjana 1, Solari, Esteban Lucas 1, McIntosh, Lachlan 4, Schachoff, Sylvia 1, Ibrahim, Tareq 2, Nekolla, Stephan G. 1, 3
Affiliations:
1- Nuklearmedizinische Klinik und Poliklinik, Klinikum rechts der Isar, School of Medicine, Technical University of Munich, Munich, Germany
2- Klinik und Poliklinik für Innere Medizin I, Klinikum rechts der Isar, School of Medicine, Technical University of Munich, Munich, Germany
3- DZHK (German Centre for Cardiovascular Research), partner site Munich Heart Alliance, Munich, Germany
4- Department of Physical Sciences, Peter MacCallum Cancer Centre, Melbourne, Australia
Villagran Asiares, Alberto
Copyright American Society of Nuclear Cardiology

## Slide 2
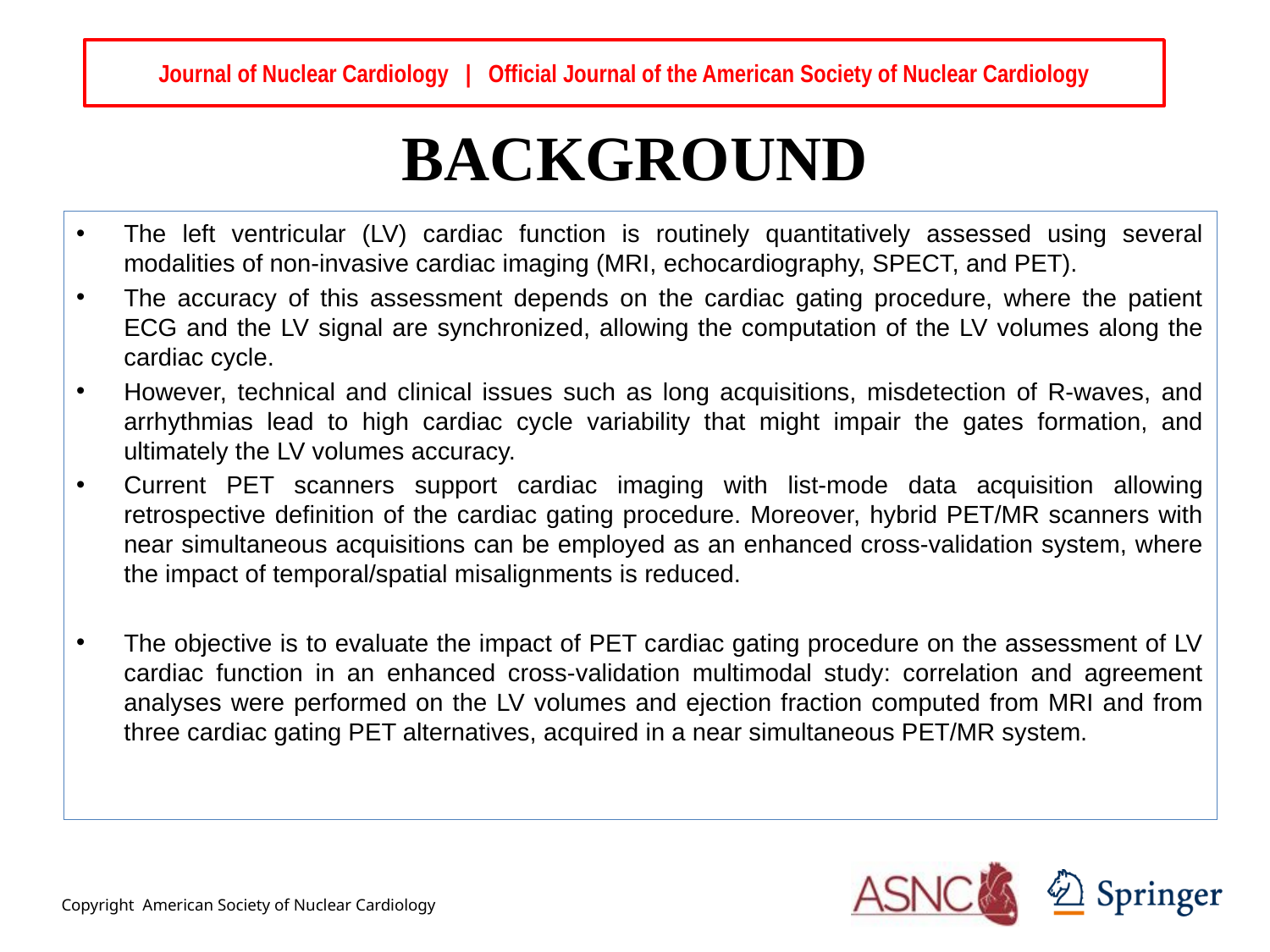

Journal of Nuclear Cardiology | Official Journal of the American Society of Nuclear Cardiology
# BACKGROUND
The left ventricular (LV) cardiac function is routinely quantitatively assessed using several modalities of non-invasive cardiac imaging (MRI, echocardiography, SPECT, and PET).
The accuracy of this assessment depends on the cardiac gating procedure, where the patient ECG and the LV signal are synchronized, allowing the computation of the LV volumes along the cardiac cycle.
However, technical and clinical issues such as long acquisitions, misdetection of R-waves, and arrhythmias lead to high cardiac cycle variability that might impair the gates formation, and ultimately the LV volumes accuracy.
Current PET scanners support cardiac imaging with list-mode data acquisition allowing retrospective definition of the cardiac gating procedure. Moreover, hybrid PET/MR scanners with near simultaneous acquisitions can be employed as an enhanced cross-validation system, where the impact of temporal/spatial misalignments is reduced.
The objective is to evaluate the impact of PET cardiac gating procedure on the assessment of LV cardiac function in an enhanced cross-validation multimodal study: correlation and agreement analyses were performed on the LV volumes and ejection fraction computed from MRI and from three cardiac gating PET alternatives, acquired in a near simultaneous PET/MR system.
Copyright American Society of Nuclear Cardiology

## Slide 3
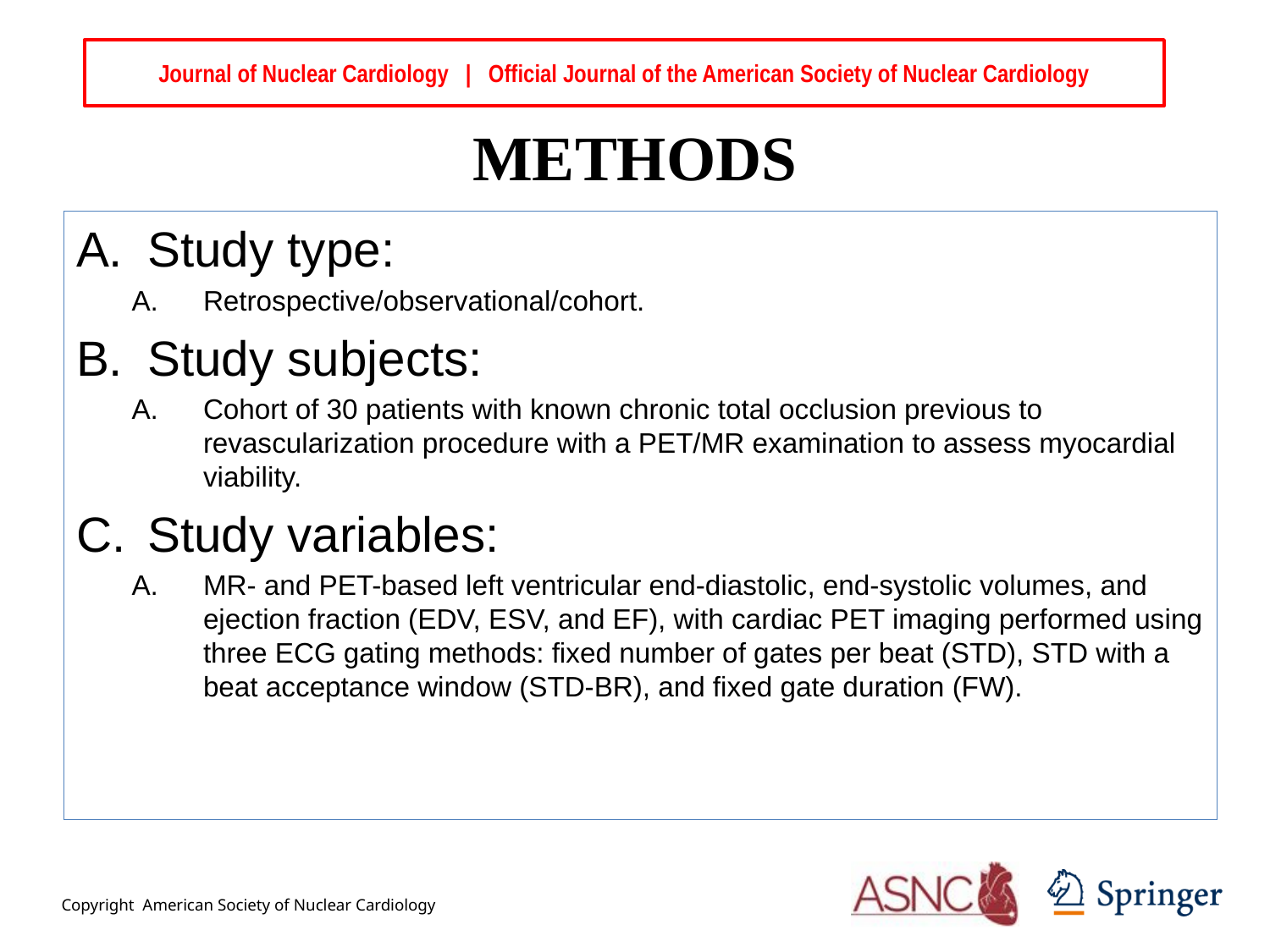

Journal of Nuclear Cardiology | Official Journal of the American Society of Nuclear Cardiology
# METHODS
Study type:
Retrospective/observational/cohort.
Study subjects:
Cohort of 30 patients with known chronic total occlusion previous to revascularization procedure with a PET/MR examination to assess myocardial viability.
Study variables:
MR- and PET-based left ventricular end-diastolic, end-systolic volumes, and ejection fraction (EDV, ESV, and EF), with cardiac PET imaging performed using three ECG gating methods: fixed number of gates per beat (STD), STD with a beat acceptance window (STD-BR), and fixed gate duration (FW).
Copyright American Society of Nuclear Cardiology

## Slide 4
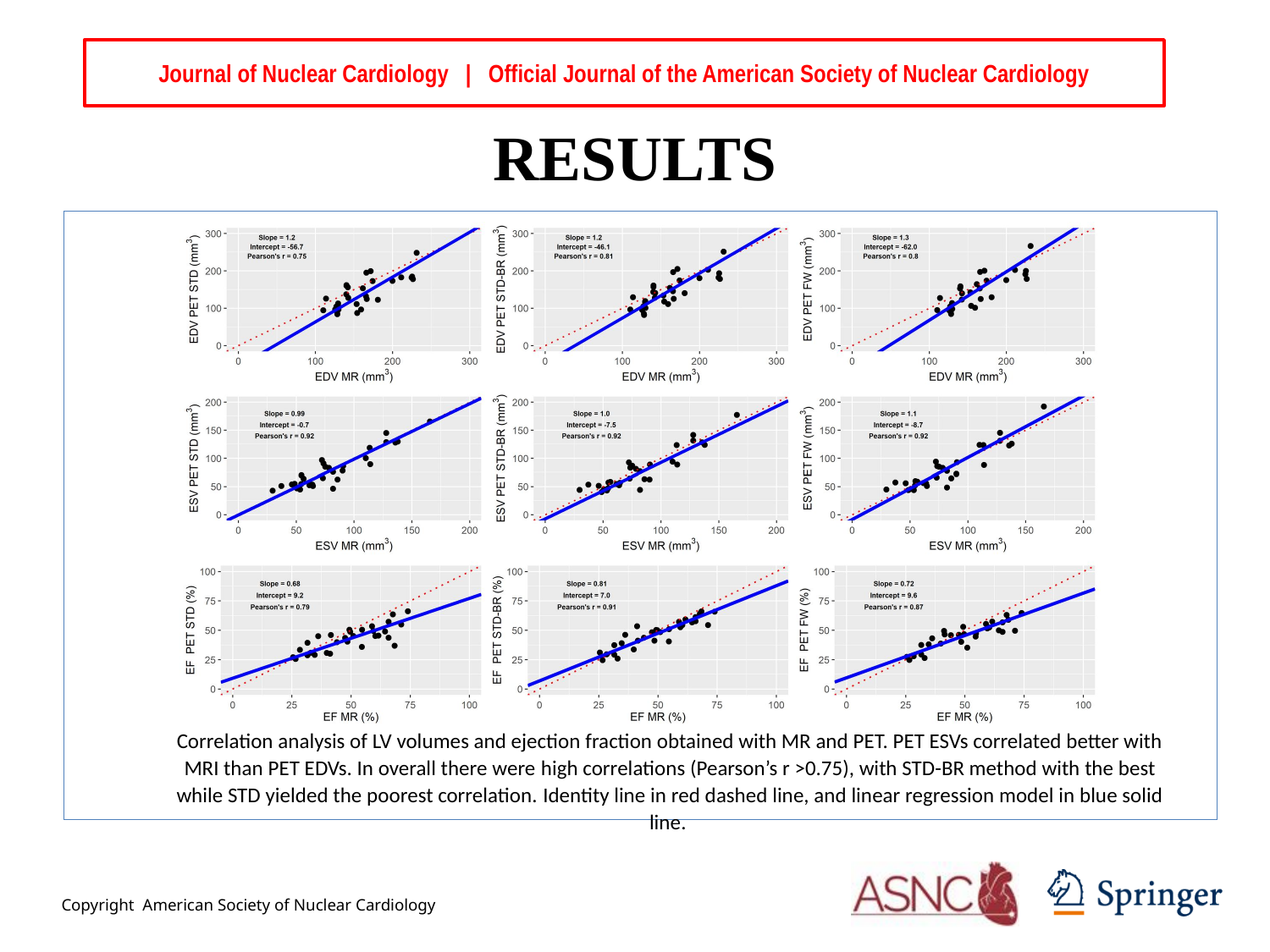

Journal of Nuclear Cardiology | Official Journal of the American Society of Nuclear Cardiology
# RESULTS
Correlation analysis of LV volumes and ejection fraction obtained with MR and PET. PET ESVs correlated better with MRI than PET EDVs. In overall there were high correlations (Pearson’s r >0.75), with STD-BR method with the best while STD yielded the poorest correlation. Identity line in red dashed line, and linear regression model in blue solid line.
Copyright American Society of Nuclear Cardiology

## Slide 5
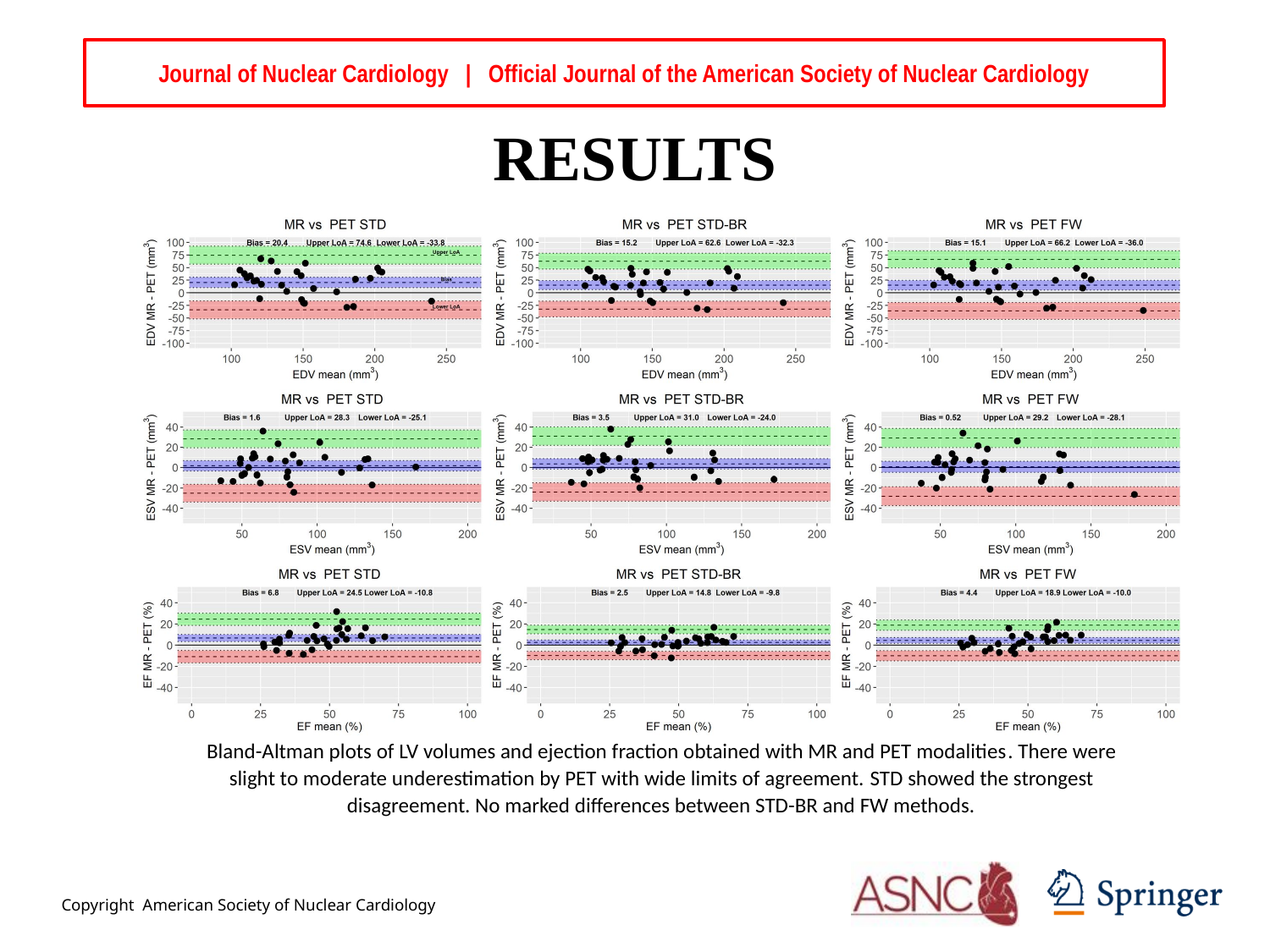

Journal of Nuclear Cardiology | Official Journal of the American Society of Nuclear Cardiology
# RESULTS
Bland-Altman plots of LV volumes and ejection fraction obtained with MR and PET modalities. There were slight to moderate underestimation by PET with wide limits of agreement. STD showed the strongest disagreement. No marked differences between STD-BR and FW methods.
Copyright American Society of Nuclear Cardiology

## Slide 6
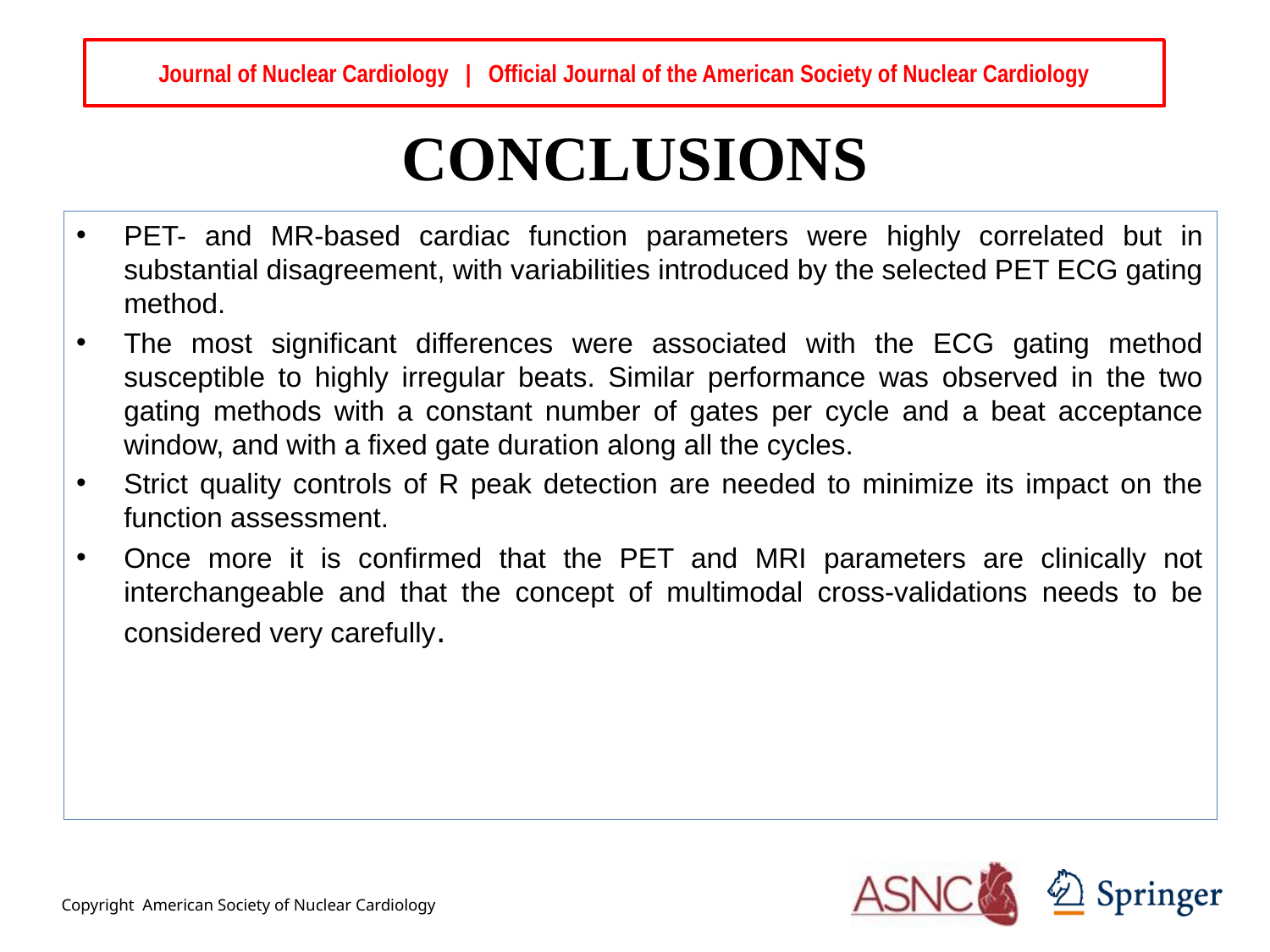

Journal of Nuclear Cardiology | Official Journal of the American Society of Nuclear Cardiology
# CONCLUSIONS
PET- and MR-based cardiac function parameters were highly correlated but in substantial disagreement, with variabilities introduced by the selected PET ECG gating method.
The most significant differences were associated with the ECG gating method susceptible to highly irregular beats. Similar performance was observed in the two gating methods with a constant number of gates per cycle and a beat acceptance window, and with a fixed gate duration along all the cycles.
Strict quality controls of R peak detection are needed to minimize its impact on the function assessment.
Once more it is confirmed that the PET and MRI parameters are clinically not interchangeable and that the concept of multimodal cross-validations needs to be considered very carefully.
Copyright American Society of Nuclear Cardiology
